# At the frontline of COVID research: an interview with Akiko Iwasaki on her groundbreaking COVID projects and advocacy for women and minorities in STEM

**DOI:** 10.1038/s42003-021-02456-9

**Published:** 2021-08-10

## Abstract

Professor Akiko Iwasaki’s research focuses on the mechanisms of immune defense against viruses at mucosal surfaces, which are a major site of entry for infectious agents. Professor Iwasaki received her Ph.D. in Immunology from the University of Toronto and completed her postdoctoral training with the National Institutes of Health before joining Yale’s faculty in 2000. She has received many awards and honors and has been a Howard Hughes Medical Institute Investigator since 2014. She was elected to the National Academy of Sciences in 2018, to the National Academy of Medicine in 2019 and to the American Academy of Arts and Sciences in 2021. Professor Iwasaki is also well known for her Twitter advocacy of women and underrepresented minorities in the science and medicine fields. In addition, Professor Iwasaki co-directs the IMPACT (Implementing Medical and Public Health Actions against Coronavirus in Connecticut) team to generate an extensive biorepository for specimens collected from patients and health care workers, as well as implementing viral testing in both groups.

**Figure:**
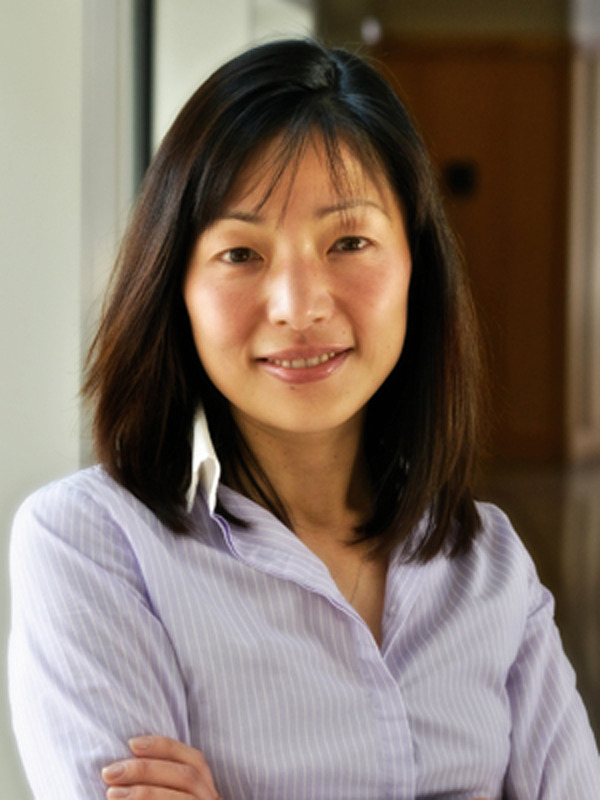
Akiko Iwasaki

Please tell us about your research interests

I am interested in virus infections and immunity. I started my career by studying how dendritic cells prime T cells in the lymph node after herpes virus infection, and the importance of innate sensor signaling in the generation of adaptive immunity. I then became interested in T cells that provide protection at the mucosa, namely the tissue-resident memory T cells, and how they are established. Based on this insight, we developed a vaccine strategy called “Prime and Pull”, where we can recruit and establish tissue resident memory T cells at the site of viral encounter. This led to a much more robust protection from genital herpes, and can also be used to treat existing virus infection to prevent recurrence of herpes lesions. In recent years, we have also invested our effort in understanding how endogenous retroelements impact our immune system. These are remnants of retroviruses that have integrated into our genome over millions of years. They are mostly damaged or silenced, but sometimes, they become aberrantly expressed in autoimmunity and cancer. We recently demonstrated that one of the endogenous retroviral envelopes can become the target of antibody attack in lupus patients. We are interested in digging deeper into the link between endogenous retroviruses and autoimmunity as well as possible use of these as vaccine antigens for cancer. We are always looking for new and improved vaccine strategies against mucosal viral infections and cancer. Since March of 2020, we have been focusing on immune response to COVID-19.

You have made so many crucial contributions to COVID-19 research. Which one are you most proud of?

It is impossible to choose, because one study leads to another and they all build on each other!

Aside from shifting focus to COVID research, how has the pandemic changed your career in the last year?

The pandemic has fundamentally changed the way I approach science and viral disease. Having focused heavily on understanding immune response in COVID patients, and knowing how important it is to study human immune response to truly understand the importance of various immune mechanisms against SARS-CoV-2, my lab is shifting to translational research in other areas of study as well. Another thing I learned over the pandemic is the importance of team science. I have learned a lot about what it takes to do human translational research as a member of a large team over the past 16 months, and I intend to keep doing more of this.

Now that we are beginning to enter a new normal, what non-COVID research are you excited to pick up again?

Even after the COVID spread is contained by vaccines, millions of people are suffering from devastating long term consequences of post-acute COVID-19 syndrome every day. We are focusing on the disease pathogenesis and based on that insight come up with rational therapy. I hypothesized that long COVID can be caused by persistent virus infection, viral remnants, or autoimmune reaction. We are now probing these hypotheses by studying blood of long haulers in collaboration with Dr. David Putrino’s group at Mt. Sinai. We are also collaborating with Drs. Harlan Krumholz, Aaron Ring and others on how vaccines might impact long COVID. Survivor Corps has reported that about 40% of long haulers experienced improvement in their symptoms, while 15% worsened. We are trying to correlate immune changes to the symptom changes to figure out what is making people feel better, and to come up with the proper therapy based on that insight. We believe this insight may also help those suffering from other post infection syndromes including myalgic encephalomyelitis/chronic fatigue syndrome (ME/CFS).

In addition to long COVID research, we are also putting back effort on our existing research program on endogenous retroelements, vaccine development, and cancer immunity.

You have been an advocate for women and mothers in STEM. What do you think needs to change to address the existing gender disparity in STEM fields and support work-life balance?

My advocacy for women and minorities in science will continue. The pandemic has unfortunately reversed some of the progress we were making on equity and inclusion. Women were disproportionately affected by the pandemic, and some had to leave science to take care of their families. We need investment to reinvigorate the workforce, and to support the fragile careers of younger scientists who are just starting their laboratories. This requires funding from the NIH and NSF. As a member of the council for the American Association of Immunologists, I get to communicate with the members of US Congress about this need, and will continue to advocate on behalf of scientists. I am hopeful that there will be bipartisan support for the proposed measures like the Research Investment to Spark the Economy Act or the RISE Act, and I am hopeful that there will be funds available to help women and younger scientists recover from the impact of the pandemic.

Aside from the post pandemic recovery measures, we still have a lot of work to do to address gender disparity. We need to continue to tackle sexual harassment, implicit bias, unequal pay, lack of access to affordable childcare, lack of opportunities and support, to name some specifics but also to fundamentally change the culture of academia so that people of any background can thrive equally to achieve their potential.

What advice would you give to young women in STEM trying to establish their independent career?

While the job market for careers in STEM also took a hit from the pandemic, many places are now reopening and recruiting new faculty and research staff. Many universities are extending the tenure clocks and providing supplemental support to restore the careers of junior investigators. I am hopeful that careers in STEM will pick up very shortly, and there will be demand for women scientists everywhere. My message to young women in STEM would be to find mentors and role models to guide them at this time of difficulty. There are so many experienced and capable senior scientists eager to help junior scientists. Having more than one mentor is also a good idea—you can get diverse opinions on a given issue and base your decision on those opinions.

*This interview was conducted by Associate Editor Karli Montague-Cardoso.*

